# DISCRIMINATION AND MINORITY STRESS IN OLDER ADULTHOOD: NEW DIRECTIONS FOR PSYCHOLOGICAL RESEARCH

**DOI:** 10.1093/geroni/igad104.1662

**Published:** 2023-12-21

**Authors:** Megan Wilson

## Abstract

The experience of marginalization can be harmful to individuals, in part because of increased experiences of discrimination (cite) and stress related to minority identities (cite). While older adults do tend to report experiencing less discrimination, past work suggests that older adults may be more impacted by experiences of discrimination compared to younger adults. Considering older adults from marginalized groups specifically, these individuals may be particularly susceptible to negative effects of discrimination related to age and other group identities that may harm their well-being and health. Given the wide-ranging negative implications of experiences of discrimination for older adults in particular, there is a need to examine potential strategies for combatting these negative effects. Thus, this symposium will examine the roles of discrimination and marginalization across aspects of health and well-being, and how these effects may differ for different marginalized groups. In addition, the symposium will discuss potential strategies and tools to help reduce harmful effects of discrimination and marginalization on older adults’ lives. First, Danielle McDuffie will present work on bereavement and gratitude for Black adults. Next, Megan Wilson will discuss nuances in the relationship between discrimination and purpose and their implications for health. Next, Lydia Ong will examine the effects of daily discrimination on cortisol reactivity. Finally, Michael Vale will discuss minority stress and loneliness for sexual minority individuals.

